# The Polysaccharides from *Balanophora harlandii* Hook. Alleviated Alcoholic Steatohepatitis via Suppressing Bile Acid Synthesis Mediated by Intestinal FXR Activation

**DOI:** 10.3390/nu18172897

**Published:** 2026-09-03

**Authors:** Li Xiao, Jingjing Chen, Wenyan Zhong, Fan Peng, Chengfu Yuan

**Affiliations:** 1 College of Basic Medical Science, China Three Gorges University, Yichang 443002, China; xiaoli-cn@163.com (L.X.); jingjingchen0924@163.com (J.C.); zwyctgu@163.com (W.Z.);; 2 Hubei Key Laboratory of Tumor Microenvironment and Immunotherapy, China Three Gorges University, Yichang 443002, China

**Keywords:** alcoholic steatohepatitis, bile acids, farnesoid X receptors, *Balanophora harlandii* Hook.

## Abstract

**Background**: Disrupted bile acid homeostasis drives alcoholic steatohepatitis (ASH) progression, which has become a new therapeutic target for ASH. This study examined the impact and underlying mechanisms of polysaccharides from *Balanophora harlandii* Hook. (BHP) on bile acid synthesis and liver damage in ASH. **Methods**: A Binge-on-Chronic alcohol exposure model was established to assess the impact of BHP on alcohol-induced liver damage and bile acid (BA) metabolism. Fexaramine and glycine-β-MCA (Gly-MCA) were also utilized to explore a crucial role of intestinal FXR signaling activity in ASH mice. In vitro, intestinal epithelial cells were stimulated with alcohol to impair FXR activity. **Results**: BHP treatment mitigated weight loss, reduced hepatic lipid accumulation and inflammation in chronic alcohol-fed mice, demonstrating a significant hepatoprotective effect against ASH. Mechanistically, BHP treatment reduced intestinal conjugated BA levels and activated FXR-FGF15 signaling, which inhibited hepatic BA synthesis and mitigated the toxic effects of hepatic BA accumulation in ASH. However, the suppression of intestinal FXR activation notably diminished the ability of BHP to regulate BA synthesis and prevent liver damage in ASH mice. In vitro, BHP treatment also activated FXR signaling in intestinal epithelial cells, counteracting alcohol-induced FXR activity damage. **Conclusions**: BHP treatment effectively alleviated liver damage in mice with chronic alcohol exposure through reducing hepatic BA synthesis. The mechanisms behind the hepatoprotective effects of BHP suggested that correcting the shift in the intestinal BA profile and/or the intestinal FXR-FGF15 signaling are the potential therapies against alcoholic liver injury.

## 1. Introduction

Patients with alcoholic steatohepatitis (ASH) frequently presented with cholestasis, a liver issue characterized by the accumulation of bile acids (BAs) [1]. Early clinical studies indicated that patients with ASH exhibited cholestasis or accumulation of BAs in the liver and systemic circulation [2], and significant intraparenchymal cholestasis independently predicted a poor short-term outcome for ASH [3]. Recent studies have shown that alcohol consumption expanded the BA pool, reduced BA excretion and shifted BAs to more hydrophobic and toxic species, contributing to hepatitis, liver fibrosis and cirrhosis, and potentially increasing the risk of hepatic tumors [4]. Hepatic BA accumulation and metabolic disorders have been shown to be an important feature and strong risk factor for ASH.

Alcohol-induced impairment of intestinal Farnesoid X receptor (FXR) signaling represented a crucial pathogenic connection between BA homeostasis and liver injury in ASH. Hepatic BA synthesis is predominantly controlled by the feedback from the intestinal FXR signaling, which produces fibroblast growth factor 19 (FGF19/mouse FGF15) to inhibit hepatic CYP7A1 activity, a crucial enzyme responsible for BA synthesis [5]. Alcohol consumption directly impaired the intestinal barrier and FXR activity, increasing permeability [6]. Chronic alcohol intake also disrupted gut microbial homeostasis to impair intestinal FXR signaling through the transformation of metabolites, especially the potent natural regulator, BAs [7,8,9].

*Balanophora harlandii* Hook. (*B. harlandii* Hook.), a well-known traditional Chinese herb from the family Balanophoraceae, is mainly found in the south-west of China [10]. As a national medicine for a long time, it has been used extensively in treating gastrointestinal bleeding, hepatitis, impotence and other diseases. Chemical analysis in Balanophoraceae has identified a variety of compounds such as flavonoids, phenylpropanoids, tannins and polysaccharides [11]. Our previous research has explored the basic composition and structure of polysaccharides from *B. harlandii* Hook. [12] and found that it has a significant inhibitory effect on inflammatory responses in both in vivo and in vitro experiments, which to some extent explains the role of *B. harlandii* Hook. in traditional medicine for treating gastrointestinal inflammatory diseases [13]. However, whether polysaccharides are the important components to the hepatoprotective effect of *B. harlandii* Hook. needs to be further explored. This study employed a chronic-binge alcohol-fed mice model to evaluate BHP’s impact on ASH and elucidated its underlying mechanism, thereby advancing drug development and application.

## 2. Methods

### 2.1. Preparation the Polysaccharides of Balanophora harlandii Hook. (BHP)

*B. harlandii* Hook. plant samples were collected from Wufeng County (Hubei, China), and were subsequently identified and stored in the Institute of Metabolic Diseases Drug Research of China Three Gorges University. In brief, the degreased *B. harlandii* Hook. was extracted with boiling water reflux (solid–liquid ratio 1:30) three times. The extract was concentrated to approximately 20% of its original volume at 55 °C and then was treated with triploid 95% alcohol at 4 °C overnight to yield the crude polysaccharide of *B. harlandii*. The crude polysaccharide was treated with dephenolization, protein removal and decolorization. Then the supernatant was separated by DEAE-52 fiber column and purified by Sephadex G-100 to obtain the polysaccharides of *B. harlandii* Hook. (BHP, marked in previous reports as BHP-W-S3, 82.71%). Subsequent studies revealed that BHP primarily consists of glucose, galactose and xylose, classifying them as relatively uniform heteropolysaccharides [12].

### 2.2. Animal Experiments and Sample Collection

Male C57BL/6 N mice, aged 8 to 10 weeks, were sourced from Laboratory Animal Center of China Three Gorges University (SCXK (E) 2022–0012) in Yichang City, Hubei Province, China. We developed chronic ASH mice using a chronic-binge alcohol feeding model [14]. Mice were randomly assigned to four groups: pair-fed group (PF), alcohol-fed group (AF), alcohol-fed with low-dose BHP (100 mg/kg/d, BHP-L), and alcohol-fed with high-dose BHP (200 mg/kg/d, BHP-H). Each group consisted of 12 mice. For four weeks, the mice were fed a Lieber-De Carli diet containing 5% ethanol (*v*/*v*) (Trophic Animal Feed High-tech Co. Ltd., Nantong, China). Both pair-fed and alcohol-fed mice were provided with the same daily caloric intake. To investigate the effects of FXR activity on ASH, mice were categorized into the following groups: pair-fed group (PF), alcohol-fed group (AF), alcohol-fed with high-dose BHP (AF + BHP), pair-fed with high-dose BHP (PF + BHP), alcohol-fed with Fexaramine (AF + Fex, 100 mg/kg/d), alcohol-fed with BHP and Glycine-muricholic Acid (AF + BHP + Gly-MCA, 10 mg/kg/d). Fexaramine and Gly-MCA were used in the last two weeks to intervene in mice through gavage. All animal experiments received approval from the Three Gorges University Animal Care and Use Committee (Ethical approval number 202401D2) and adhered to National Institutes of Health guidelines.

### 2.3. Tissue Staining and Immunofluorescence Analysis

Frozen sections of the liver were used for oil red staining. Paraffin embedding and hematoxylin–eosin staining were performed on formalin-fixed liver and ileum samples following standard protocols. For the immunofluorescence assay, paraffin sections or cell slides were blocked with 5% goat serum and 0.3% Tween-20 in PBS to prevent nonspecific binding, followed by an overnight incubation at 4 °C with rabbit anti-mouse occludin antibodies (Proteintech Group, Inc., Chicago, IL, USA, 1:100). Following washing, the sections were incubated in the dark at room temperature for 2 h with Cy3-conjugated goat anti-rabbit IgG (Boster Biological Technology, Wuhan, China, 1:1000). Cover slips were mounted on slides using fluoroshield mounting medium with DAPI (SouthernBiotech, Homewood, AL, USA, 1:1000). The images were observed and captured using an Olympus BX53 microscope (Shinjuku, Tokyo, Japan).

### 2.4. Determination of ALT, TC, TG and Total Bile Acids

ALT and AST activities, as well as total glyceride, cholesterol, and bile acid levels in serum or liver tissues, were measured using Biochemical kits from Nanjing Jiancheng Bioengineering (Nanjing, China). Mouse FGF15 ELISA KIT were purchased from Invitrogen (Carlsbad, CA, USA). Fecal bile salt hydrolase (BSH) activity was assessed by homogenizing fecal samples in PBS (pH 7.4, 0.5 g/1 mL) and centrifuging them to obtain the supernatant. Then, the samples were used to detect BSH activity using ELISA kits (G0933W, Grace Biotechnology, Suzhou, China). For convenient analysis, values below the detection threshold were treated as zero.

### 2.5. Real-Time Quantitative PCR (qRT-PCR)

RNA was extracted from mouse liver and ileum tissues using Trizol reagent (Takara Bio Inc., Kusatsu, Japan) according to the manufacturer’s instructions. cDNA was then synthesized from 3 mg of total RNA utilizing M-MLV Reverse Transcriptase (Promega Corporation, Madison, WI, USA). The real-time quantitative PCR experiment was carried out using the analytikjena qTOWER3G real-time PCR instrument. PCR data were normalized to GAPDH mRNA expression employing the 2^−CΔΔt^ method. Supplementary Table S1 lists the primers utilized in the experiments.

### 2.6. Western Blot

Protein was extracted from mouse liver or ileum tissues, and quantified via the BCA method. The equivalent protein was separated using SDS-PAGE gel and then transferred to the polyvinylidene difluoride (PVDF) membranes. Membranes were blocked with 5% milk for 2 h and then incubated overnight at 4 °C with the primary antibody. The strips were incubated for 2 h at 37 °C with a secondary antibody conjugated to HRP, diluted at 1:4000. Images were captured using ECL chemiluminescence and a Bio-Rad gel imaging system, with band quantification done via ImageJ 1.54g software. β-actin is used as an internal reference. Data are presented as fold change relative to the control group. The experiments employed the following antibodies: FXR (1:1000, Abconal, Wuhan, China), FGF15 (1:500, Abcam, Cambridge, UK), CYP7A1 (1:1000, Abcam), ASBT (1:500, Bioss), Cyp27A1 (1:500, abconal), FGF19 (1:500, Abcam), Occludin (1:1000, Proteintech, Rosemont, IL, USA), NTCP (1:1000, Abcam), BSEP (1:500, Bioss, Beijing, China), β-actin (1:4000, Proteintech) and HRP-labeled secondary antibody (1:8000, Proteintech).

### 2.7. Bile Acids Assay

Ultra-performance liquid chromatography–mass spectrometry (LC-MS) was employed to quantify BAs in mouse ileal contents. Briefly, the samples were homogenized with 2 volumes of deionized water (*w*/*v*) on ice. The homogenized mixture was diluted with 80% methanol and centrifuged at 15,000 rpm, 4 °C for 15 min. Then the supernatant was evaporated to dryness under nitrogen. The dried residue was reconstituted in 50 μL 50% methanol containing the mixed internal standard and centrifuged at 15,000 rpm, 4 °C for 15 min to obtain the prepared sample. The deuterium-labeled BAs were used as internal standards (ISs) for quantification. BAs quantities were analyzed by an Agilent 1290 Infinity II UHPLC system coupled to a 6470A Triple Quadrupole mass spectrometry (Santa Clara, CA, USA).

### 2.8. Cell Culture

Caco-2 and MC38 cell lines, sourced from the Type Culture Collection of Chinese Academy of Science’s, were cultured in DMEM (including 10% fetal bovine serum) at 37 °C in a 5% CO_2_ atmosphere. Caco-2 or MC38 cells were exposed to alcohol (200 nM), BHP (50, 100 μg/mL) or T-β-MCA (10 µM) for 24 h to assess the effect of BHP on FXR activity. FXR and FGF15 (or FGF19) expressions were subsequently measured.

### 2.9. Statistical Analysis

GraphPad Prism 10 (GraphPad Software Inc., San Diego, CA, USA) was used for statistical analyses. Data are presented as mean ± standard deviation (SD). Data normality was assessed by the Shapiro–Wilk test. Homogeneity of variances was evaluated using Levene’s test. For comparisons between two groups, Student’s *t*-test (parametric) or Mann–Whitney U test (non-parametric) was applied. For multiple group comparisons, one-way ANOVA (parametric) or Kruskal–Wallis test (non-parametric) was used, followed by Tukey post hoc analysis. A *p*-value below 0.05 was considered statistically significant.

## 3. Results

### 3.1. BHP Treatment Ameliorated Liver Injury in Chronic Alcohol-Fed Mice

**Figure 1:**
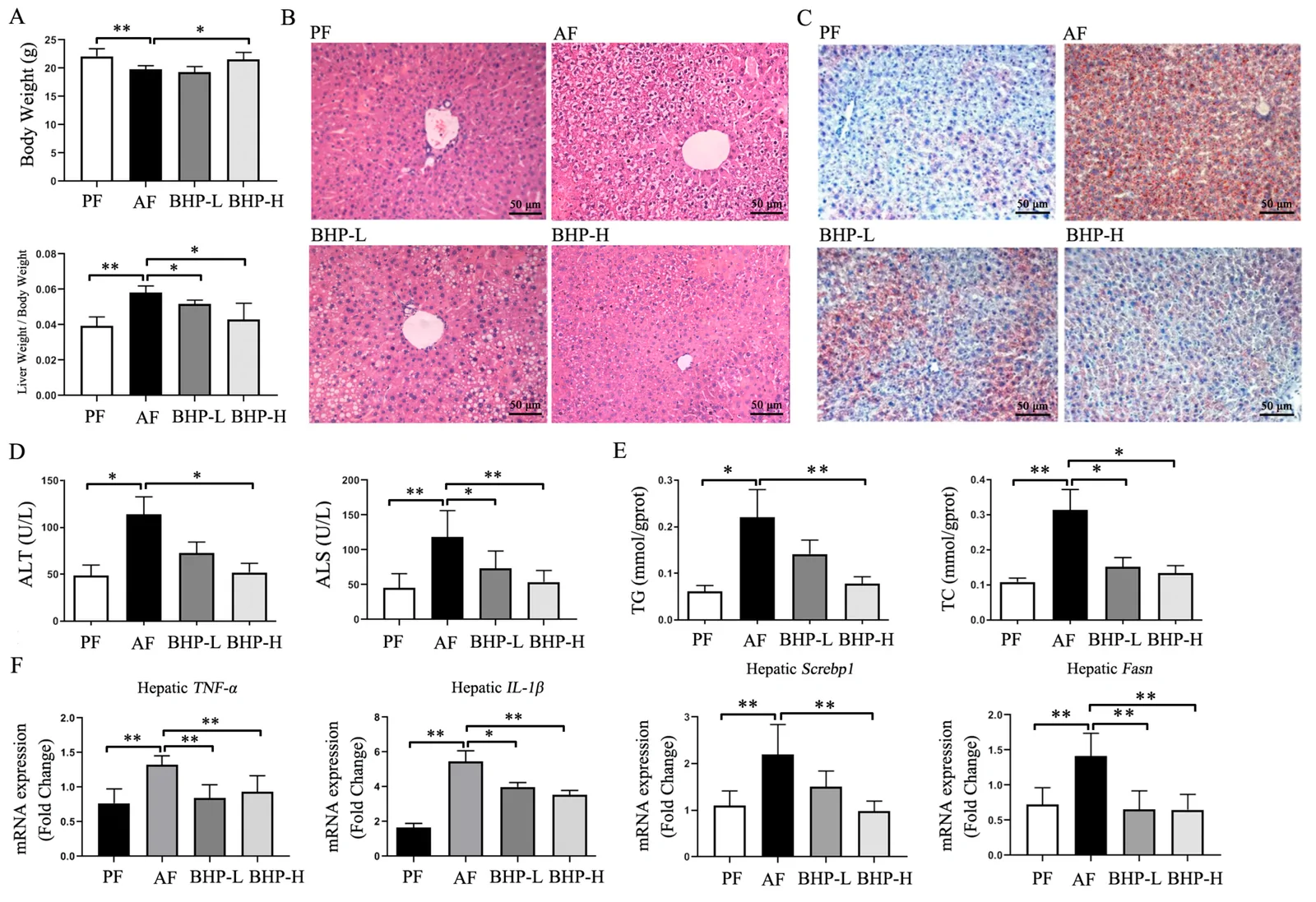
BHP treatment ameliorated hepatic injury in mice exposed to chronic alcohol. (**A**) Body weight, liver/body weight ratio; (**B**) liver HE staining (×200); (**C**) liver oil red O staining (×200); (**D**) serum ALT, AST activities; (**E**) hepatic triglyceride (TG) and total cholesterol (TC) levels; (**F**) hepatic mRNA expression of *TNF-α*, *IL-1β*, *SREBP1*, *Fasn*. Data are presented as means ± SD (*n* = 8–10). PF, paired fed; AF, alcohol fed; BHP-L, AF + BHP-L; BHP-H, AF + BHP-H; * *p* < 0.05; ** *p* < 0.01.

Mice fed with a 4-week chronic alcohol-liquid diet exhibited the characteristics consistent with an ASH model, including notable liver steatosis, vacuolation, and excessive inflammatory cell infiltration. BHP treatment effectively prevented body weight loss and inhibited the increase in liver/body weight ratio of mice with chronic alcohol-liquid diet (Figure 1A). After BHP administration, liver pathological changes induced by chronic alcohol consumption, such as hepatic lipid accumulation, inflammatory infiltration, and hepatocellular injury, were markedly alleviated in ASH mice (Figure 1B,C and Figure S1). Alcohol exposure in mice led to elevated serum ALT and AST activities, which were dose-dependently reversed by BHP intervention (Figure 1D). This intervention also reduced total triglyceride and cholesterol levels in the liver of ASH mice (Figure 1E). Moreover, alcohol-liquid diet notably elevated hepatic mRNA expressions of *TNF-α*, *IL-1β*, *Screbp1*, and *Fasn* in mice exposed to alcohol (Figure 1F). The findings showed that BHP treatment effectively mitigated hepatic steatosis and excessive inflammatory reaction in chronic alcohol-fed mice.

### 3.2. BHP Treatment Changed Intestinal Bile Acid Profile in ASH Mice

**Figure 2:**
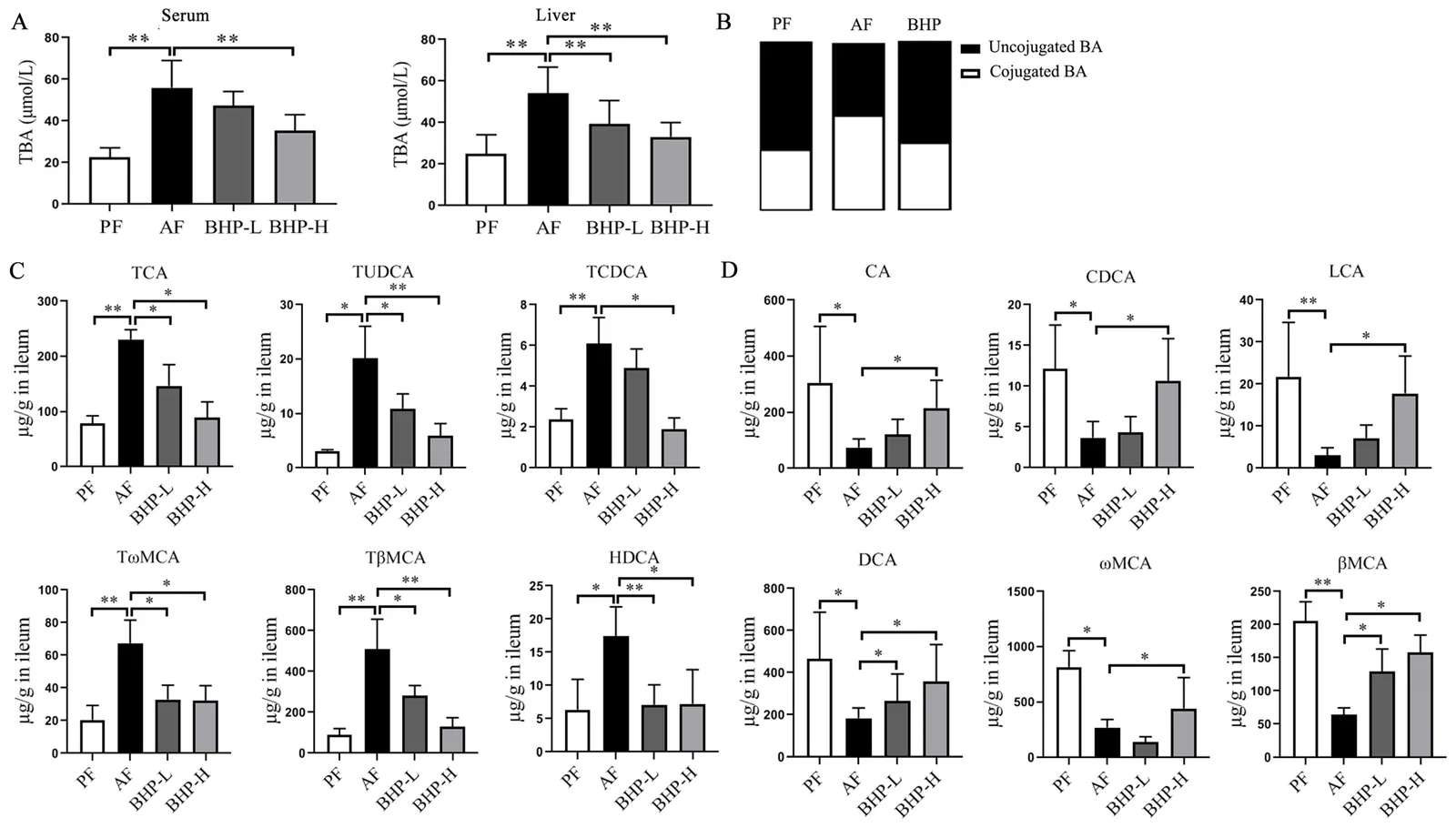
BHP treatment altered the ileal bile acid metabolism in ASH mice. (**A**) Total bile acid contents in serum, liver and ileum; (**B**) the proportion of unconjugated and conjugated bile acid in the ileum; (**C**) the major conjugated bile acid contents in ileum; (**D**) the major unconjugated bile acid contents in ileum. Data are presented as means ± SD (*n* = 5). PF, paired fed; AF, alcohol fed; BHP-L, AF + BHP-L; BHP-H, AF + BHP-H; * *p* < 0.05; ** *p* < 0.01.

Alcohol intake led to the expansion of the BA pool and a shift in BA profile [15]. Chronic alcohol exposure significantly increased the levels of total BAs in serum and liver of mice, while BHP treatment at both low and high doses notably reduced these levels (Figure 2A). Except for total BA levels, the modifications of BA composition in ileum are presented in Figure S2. Along with changes in total BA levels, chronic alcohol consumption resulted in a notable rise in the ratio of conjugated BAs and a reduction in unconjugated BAs in ileum contents, compared to the paired-fed group. BHP supplementation countered the alcohol-induced changes in the types of BAs in the ileum of mice (Figure 2B), suggesting a beneficial effect brought by the growth of BSH-producing bacteria in ASH mice promoted by BHP treatment.

Conjugated BAs, especially tauro-conjugated BAs, are generally more potent inhibitors of FXR activity relative to unconjugated BAs [16]. The further analysis revealed that alcohol intake significantly increased the levels of tauro-α-muricholic acid (T-α-MCA), T-β-MCA, tauroursodeoxycholic acid (TUDCA) and hyodeoxycholic acid (HDCA) in the ileum (Figure 2C), and decreased the contents of α-muricholic acid (α-MCA) and chenodeoxycholic acid (CDCA) (Figure 2D). However, BHP significantly resisted the conversion effect of alcohol on ileal BAs, decreased the contents of intestinal T-α-MCA, T-β-MCA and TUCDA, and increased CDCA, lithocholic acid (LCA), α-MCA and ω-MCA levels. These data indicated that BHP treatment induced a reduction of tauro-conjugated BA accumulation, especially T-α-MCA, T-β-MCA and TUCDA, the inhibitors of intestinal FXR activity, in alcohol-fed mice.

The analysis of gut microbiota at the genus level revealed that the addition of BHP significantly increased the abundance of Lactobacillus, Bifidobacterium, and Bacteroides, as high BSH-producing bacteria, in mice fed an alcoholic diet. In contrast, Prevotella and Muribaculum, as low BSH-expressing bacteria, were not significantly altered by BHP treatment (Figure S3). Bile salt hydrolase (BSH) was produced by intestinal bacteria, the primary enzyme responsible for the balance between conjugated and unconjugated BAs in the gut [17]. The BSH activities in ileal contents of mice with alcohol diet were significantly reduced, which were enhanced after BHP supplementation (Figure S4). It is speculated that BHP may have a regulatory effect on the BSH-producing microbiota.

### 3.3. BHP Treatment Enhanced Intestinal FXR-FGF15 Signaling in ASH Mice

**Figure 3:**
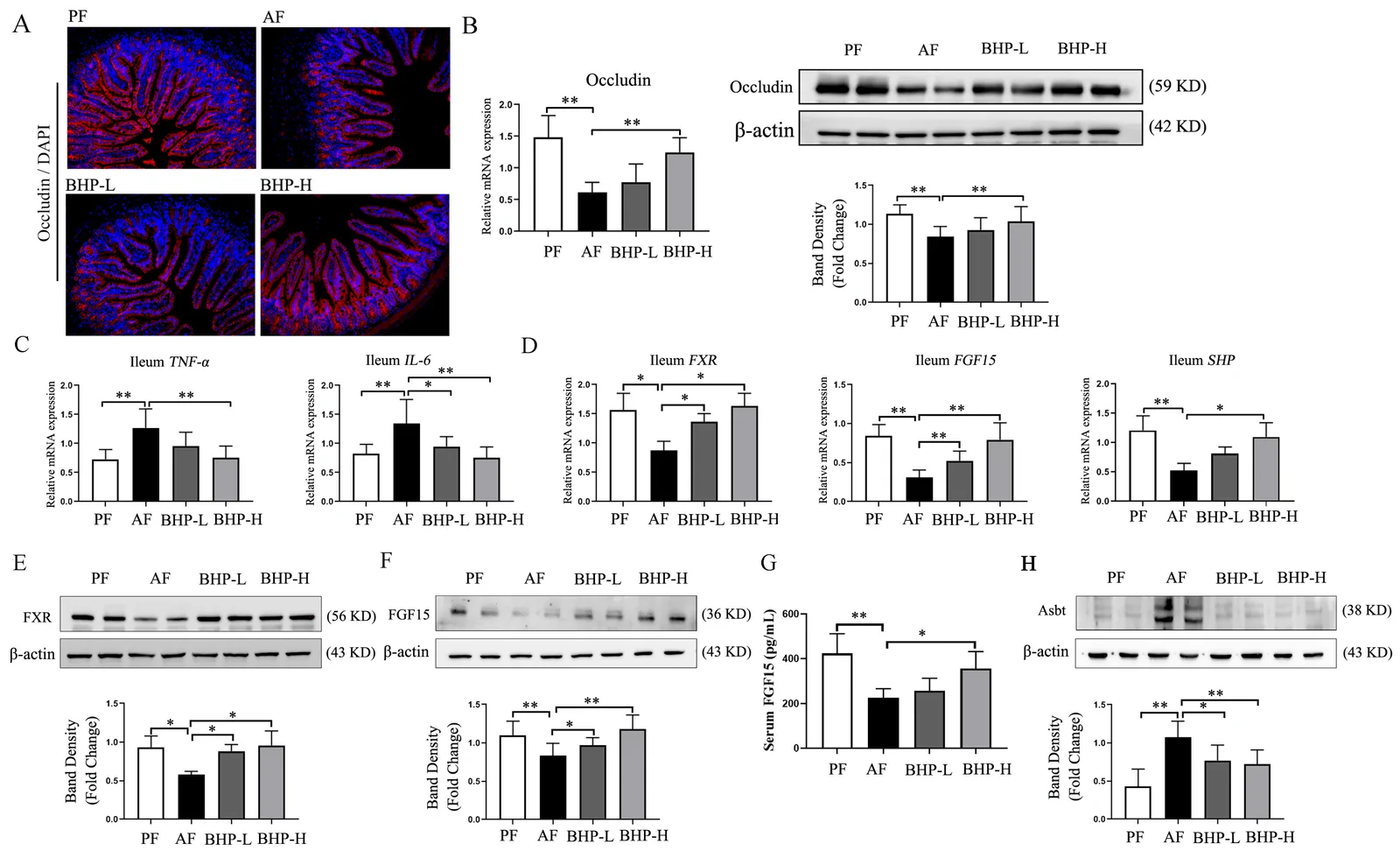
BHP treatment enhanced intestinal FXR-FGF15 signaling in mice with ASH. (**A**) Representative images of Occludin staining (red) in ileum; (**B**) Occludin mRNA and protein expression levels in ileum; (**C**) *TNF-α*, *IL-1β* mRNA expressions in ileum; (**D**) *FXR*, *FGF15* and *SHP* mRNA expressions in ileum; (**E**) ileal FXR protein expression; (**F**) ileal FGF15 protein expression; (**G**) Serum FGF15 levels; (**H**) ileal Asbt protein expression. Data are presented as means ± SD (*n* = 8). PF, paired fed; AF, alcohol fed; BHP-L, AF + BHP-L; BHP-H, AF + BHP-H; * *p* < 0.05; ** *p* < 0.01.

Chronic alcohol consumption disturbs intestinal flora, weakens barrier function, and raises permeability [18]. Despite alcohol challenge, BHP treatment improved the disorder of colonic epithelial cells, repaired the damage in intestinal mucosal villi and reduced inflammatory cell infiltration (Figure 3A). BHP treatment also increased occludin expression, an ileal midgut tight junction protein and reduced *TNF-α*, *IL-1β* mRNA levels in ASH mice (Figure 3B,C). Notably, the administration of BHP markedly decreased serum LPS levels in mice with chronic alcohol exposure (Figure S5), indicating a protective impact on intestinal barrier function.

Hepatic bile acid synthesis is primarily regulated by feedback from the intestinal FXR-FGF15 signaling pathway. Chronic alcohol consumption markedly reduced the mRNA and protein levels of FXR and FGF15 in the mouse ileum, but BHP supplementation restored their levels (Figure 3D–F). Additionally, BHP treatment normalized the expression of ileal *SHP* mRNA in mice with chronic alcohol exposure, indicating activation of intestinal FXR signaling in ASH mice. Correspondingly, the application of BHP successfully maintained serum FGF15 levels in chronic alcohol-fed mice (Figure 3G). Moreover, alcohol consumption has been shown to affect BA transport and reabsorption in the intestine, exacerbating dysregulation of BA metabolism. BHP supplementation significantly reduced the expression of the bile acid reabsorption-related protein ASBT in the ileum of chronic alcohol-fed mice, indicating its involvement in modulating intestinal BA reabsorption (Figure 3H). We speculated that this effect of BHP may be related to the restoration of FXR activity, but the detailed mechanism still needs further studies. Combined with these findings, these data indicated that BHP treatment enhanced intestinal FXR signaling in ASH mice, which was associated with its action to reduce generation of intestinal tauro-conjugated BAs.

### 3.4. BHP Treatment Suppressed Hepatic Bile Acid Synthesis in Mice with Chronic ASH

**Figure 4:**
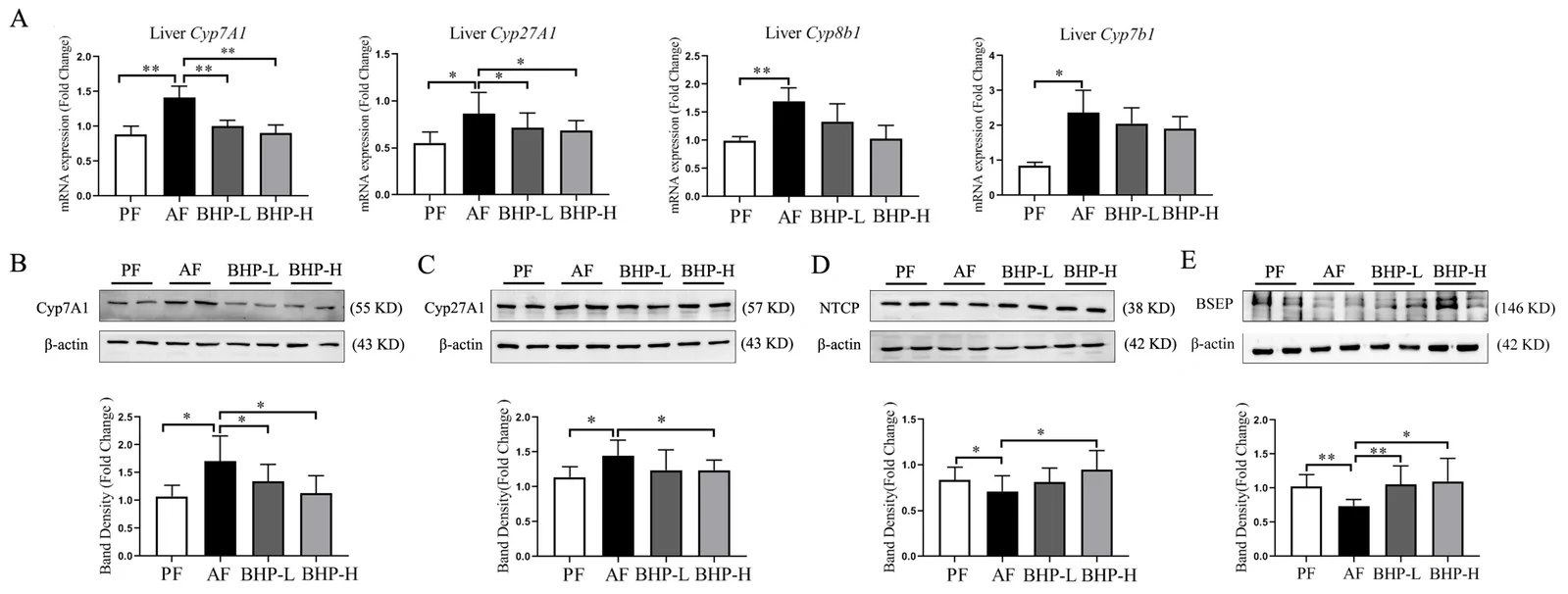
BHP treatment reduced hepatic bile acids synthesis in ASH mice. (**A**) mRNA expressions of *Cyp7a1*, *Cyp8b1*, *Cyp27A1*, *Cyp7b1* in liver; (**B**,**C**) protein expressions of Cyp7a1, Cyp27A1 in liver; (**D**,**E**) protein expression of NTCP, BSEP in liver. Data are presented as means ± SD (*n* = 8–10). PF, paired fed; AF, alcohol fed; BHP-L, AF + BHP-L; BHP-H, AF + BHP-H; * *p* < 0.05; ** *p* < 0.01.

It has been known that intestinal FXR signaling inhibits hepatic BA synthesis through FGF15 feedback [19]. Chronic alcohol consumption significantly elevated mRNA and protein expressions of Cyp7A1 and Cyp27A1 in liver, as the main rate-limiting enzymes for BAs synthesis, in mice. This increase was notably suppressed by BHP treatment (Figure 4A–C), while BHP treatment did not significantly alter *Cyp8b1* and *Cyp7b1* mRNA expression in the livers of ASH mice. Alcohol consumption resulted in lowered expression levels of hepatic NTCP and BSEP, BA transport-associated proteins, which facilitate BA transport in mice. BHP administration notably mitigated the decrease in hepatic NTCP and BSEP expression in mice with ASH (Figure 4D,E). These indicated that BHP treatment boosted intestinal FXR-FGF15 signaling, which inhibited hepatic BA synthesis and promoted BA transport in chronic alcohol-exposed mice.

### 3.5. Blocking Intestinal FXR Activation Eliminated the Hepatoprotective Effect of BHP on ASH Mice

**Figure 5:**
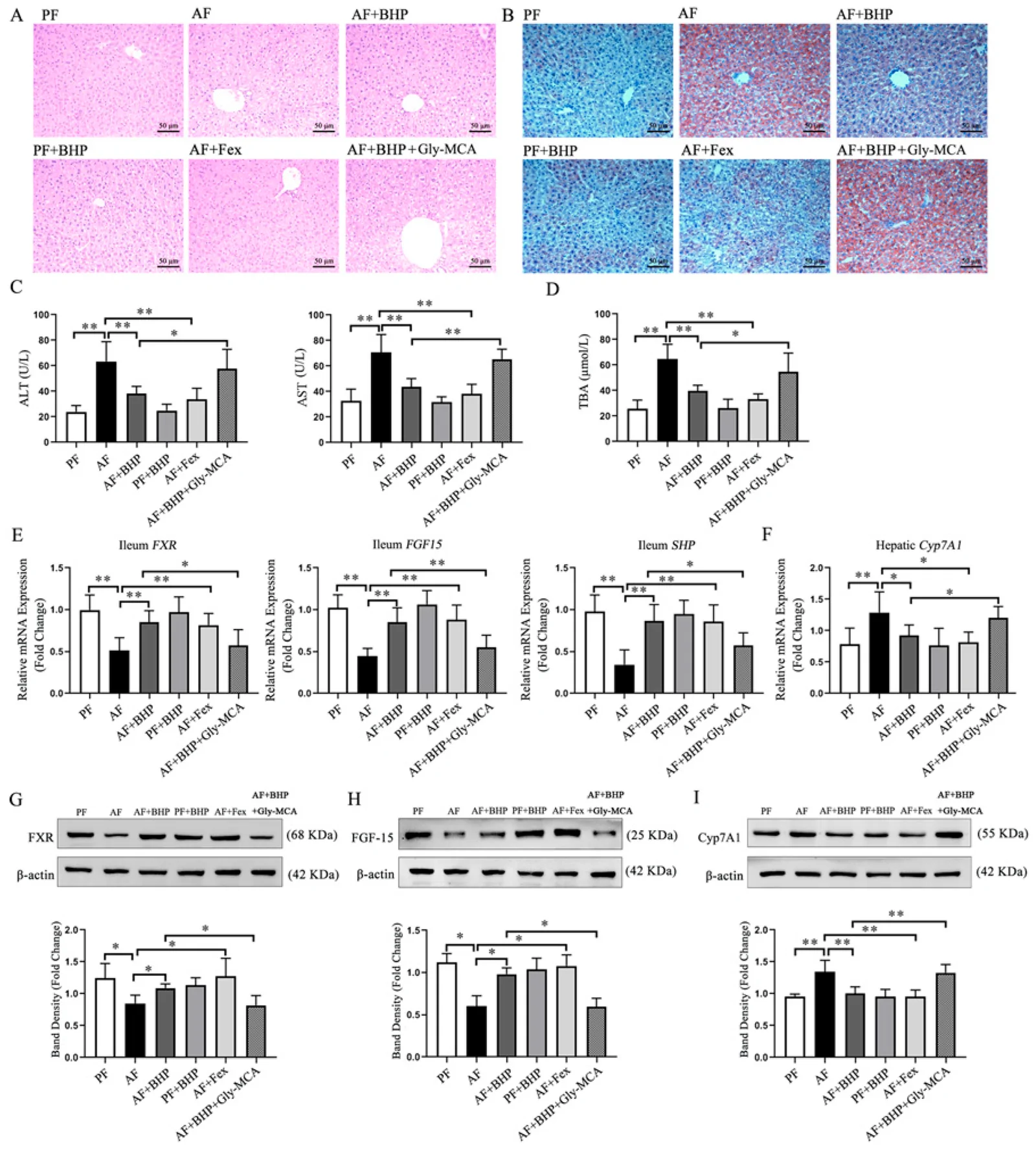
The suppression of FXR activation abolished the protective effect of BHP treatment on chronic ASH mice. (**A**) Liver HE staining; (**B**) liver oil red staining; (**C**) serum ALT, serum AST activity; (**D**) hepatic total bile acid; (**E**) *FXR*, *FGF15*, *SHP* mRNA expressions in ileum; (**F**) *Cyp7A1* mRNA expression in liver; (**G**,**H**) protein expression of FXR, FGF-15 in ileum; (**I**) protein expression of Cyp7A1 in liver. Data are presented as means ± SD (*n* = 8–10). PF, paired fed; AF, alcohol fed; Fex, Fexaramine; Gly-MCA, glycine-β-muricholic acid; * *p* < 0.05; ** *p* < 0.01.

The data presented above indicated that intestinal FXR-FGF15 signaling is pivotal in regulating the effects of BHP on BA metabolism. Consequently, we hypothesized that inhibiting intestinal FXR signaling would negate the beneficial effects of BHP in ASH mice. To elucidate this, alcohol-fed mice were given either gut-specific FXR agonist Fexaramine or FXR inhibitor glycine-β-muricholic acid (Gly-MCA). The findings demonstrated that the suppression of intestinal FXR activity notably diminished the positive impacts of BHP treatment on liver cell damage and lipid accumulation, as evidenced by increased ALT and AST activity in ASH mice (Figure 5A–C). Gly-MCA nearly nullified the protective effect of BHP in counteracting the decrease in ileal FXR and FGF15 expressions in ASH mice, leading to increased hepatic Cyp7A1 expressions and hepatic BA accumulation (Figure 5D).

In contrast, Fexaramine exhibited hepatoprotective effects in mice exposed to chronic alcohol, which was associated with the activation of intestinal FXR signaling as expected (Figure 5E–H). Intestinal FXR signal activation substantially decreased hepatic Cyp7A1 activity, prevented hepatic bile acid synthesis, and alleviated hepatic steatosis and serum ALT activity in mice exposed to alcohol (Figure 5F,I). The data suggested that blocking intestinal FXR signaling significantly impaired the hepatoprotective effects of BHP treatment against BA-induced liver injury in ASH mice. Moreover, hepatic FXR activation is involved in hepatic carbohydrate and lipid metabolism as well as inflammatory responses. Chronic alcohol intake reduced hepatic FXR protein expression, which was restored following BHP treatment (Figure S6).

### 3.6. BHP Induced FXR Activity and Counteracted Alcohol-Induced Damage to FXR Activity in Intestinal Epithelial Cells

**Figure 6:**
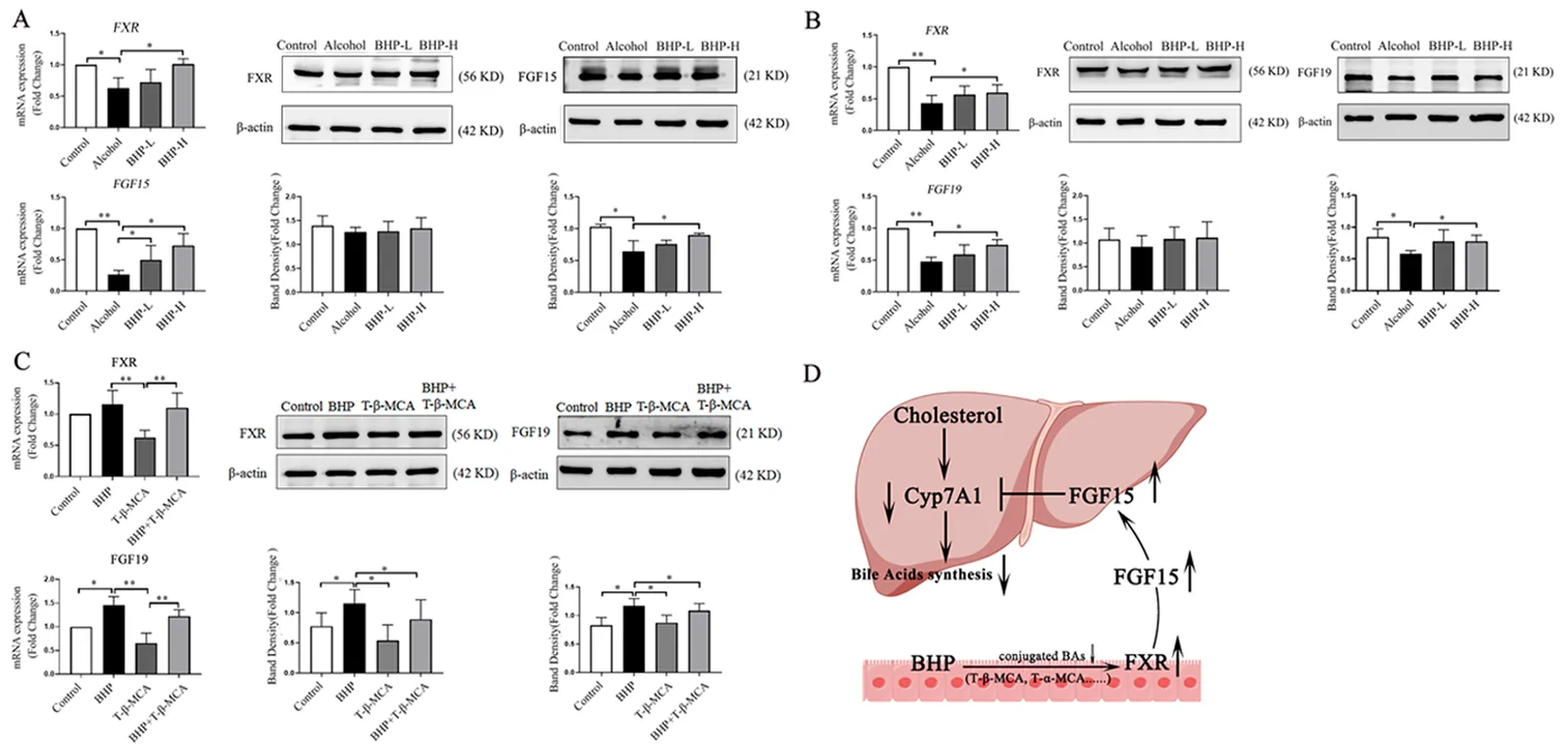
BHP enhanced FXR activation in alcohol-exposed intestinal epithelial cells. MC38 cells or Caco-2 cells were treated with alcohol (200 nM), BHP (50 μg/mL or 100 μg/mL), or TβMCA (10 µM) for 24 h. (**A**) mRNA and protein expressions of FXR, FGF15 in MC38 cells; (**B**) mRNA and protein expressions of FXR, FGF19 in Caco-2 cells. (**C**) The protein expressions of FXR, FGF19 in Caco-2 cells. (**D**) A proposed model of BHP prevention against alcohol-induced liver injury via the regulation of bile acid metabolism (by Figdraw 2.0). Data are presented as means ± SD (*n* = 3–5). * *p* < 0.05; ** *p* < 0.01.

To assess BHP’s effect on FXR activity, intestinal epithelial MC38 or Caco-2 cells were directly exposed to alcohol, with or without BHP supplementation. The results found that a 24 h alcohol treatment slightly decreased FXR protein expression in MC38 and Caco-2 cells, while significantly reducing FGF15/FGF19 protein production. This decline was reversed by the intervention of BHP (Figure 6A,B), indicating its protective effect on alcohol-induced FXR activity damage in intestinal epithelial cells. Further, Caco-2 cells were treated with BHP, with or without the FXR antagonist T-β-MCA to assess FXR activity. Interestingly, BHP treatment induced the mRNA and protein expressions of FXR and FGF19 in Caco-2 cells, exhibiting an activation effect on FXR signaling. Even in the presence of T-β-MCA, BHP still reversed the reduction of FXR and FGF19 expressions in intestinal epithelial cells (Figure 6C). These indicated that BHP induced FXR activity and counteracted alcohol-induced damage to intestinal FXR-FGF15 signaling in intestinal epithelial cells, by which BHP exerted an inhibitory effect on hepatic BA synthesis in alcohol-fed mice.

## 4. Discussion

Timely intervention and treatment during the ASH stage are the key to preventing irreversible liver lesions, such as fibrosis and cirrhosis. However, effective pharmacological treatments for ASH remain to be thoroughly investigated [20]. *B. harlandii* Hook., a traditional medicinal plant with a long history of use, has demonstrated extensive pharmacological effects on viral infections, inflammation, and tumors [21]. The study revealed that BHP, a natural polysaccharide, functioned as an intestinal microbial regulator to coordinate the feedback inhibition of intestinal FXR-FGF15 signaling and hepatic BA synthesis in ASH mice. BHP intervention effectively mitigated alterations in the gut bile acid profile of ASH mice and enhanced intestinal FXR-FGF15 signaling, resulting in reduced hepatic BA synthesis and protection against alcohol-induced liver injury.

ASH is usually accompanied by disturbances in BAs metabolism. Research has indicated that the hepatic BA synthesis was upregulated regardless of excessive BAs, due to the disruption of BA metabolism-related signaling pathways, which aggravated ASH [22,23]. The impaired regulatory axis in ASH is the gut–liver feedback loop that governs de novo BA synthesis in the liver, primarily involving intestinal FXR signal [24]. Both global FXR knockout [25] and intestine-specific FXR deficiency [26] have been found to have altered BA pool composition in mice.

Hepatocyte-specific FXR deletion did not influence BA repertoire or BA synthase activity, highlighting a dominant role of intestinal FXR activity in regulating hepatic BA synthesis [27]. Fexaramine, a gut-specific FXR agonist, was observed to reduce hepatic BA levels and alleviate chronic alcohol-induced liver injury and steatosis in ASH mice [9]. Consistent with previous reports, the current investigation showed that alcohol feeding significantly impaired intestinal FXR activity, diminished the negative feedback regulation of FGF15 on liver Cyp7A1, and increased hepatic bile acid synthesis, leading to increased BA levels in both liver and serum in mice. The use of BHP effectively countered the inactivation of intestinal FXR-FGF15 signaling in mice treated with chronic alcohol feeding, which inhibited the enhancement of hepatic bile acid synthesis in ASH mice, showing an antagonistic effect on FXR activity impairment. The hepatoprotective effect of BHP in ASH is similar to that observed with the intestinal FXR agonists Fexaramine. However, the protective effects of BHP on liver injury and BA synthesis in chronic alcohol-fed mice were nearly abolished by the administration of Gly-MCA, a specific intestinal FXR antagonist. These findings suggested that the inhibitory effect of BHP on BA biosynthesis is mainly dependent on the intestinal FXR activity of ASH mice. BHP treatment had a regulatory effect on intestinal FXR signal-mediated hepatic BAs synthesis. The effect of BHP was confirmed by that BHP treatment significantly increased the expression of FGF19 in intestine epithelial Caco-2 cells under alcohol stimulation in vitro. Cell experiments revealed that BHP alleviated the alcohol-induced damage of FXR activity in intestinal epithelial cells, indicating its potential to resist alcohol-related damage to FXR activity. However, as a macromolecule formed by the polymerization of different monosaccharides, the pharmacological mechanism of BHP in regulating intestinal FXR activity needs to be further explored.

Bile acids are endogenous ligands of FXR. CDCA is regarded as the most potent natural FXR agonist in primary BAs. T-β-MCA acts as a strong antagonist of intestinal FXR activity, and T-α-MCA and TCDCA exhibit similar properties [28]. Normally, conjugated BAs were weak FXR agonists, while their unconjugated forms exhibited higher potency [29]. In addition to inhibiting total hepatic BA levels, BHP treatment also significantly altered the profile of intestinal BAs, especially affecting the ratio of conjugated to unconjugated BAs. BHP supplementation markedly reduced the contents of conjugated BAs in the intestine of alcohol-exposed mice, especially taurine-conjugated BAs, T-β-MCA, T-α-MCA, TCDCA, and TUDCA. It has been found that a decrease in BSH-producing bacteria in chronic alcohol-fed mice increased taurine conjugated BAs, which blocked intestinal FXR activity and consequently reduced feedback inhibition on hepatic BA synthesis [30]. Furthermore, the intestinal BSH activities of mice with alcohol diet were significantly reduced, while they were enhanced after BHP addition (Figure S3). BHP may exert a regulatory effect on the BSH-producing microbiota, thereby modulating intestinal FXR activity. However, the specific mechanism still needs further study.

## 5. Conclusions

The study demonstrated that BHP treatment effectively inhibited hepatic BA synthesis by promoting the intestinal FXR-FGF15 signaling pathway and increased BA excretion, thereby mitigating chronic alcohol-induced liver injury (Figure 6D). The findings suggested BHP had the potential to treat alcoholic liver injury.

## Data Availability

The original contributions presented in this study are included in the article/Supplementary Materials. Further inquiries can be directed to the corresponding author.

## Supplementary Materials

The following supporting information can be downloaded at: https://www.mdpi.com/article/10.3390/nu18172897/s1, Table S1: Primers; Figure S1: BHP treatment significantly reduces the degree of hepatic steatosis in mice exposed to alcohol; Figure S2: Heatmap of bile acids levels in mice ileal content; Figure S3: BHP treatment increased the abundance of BSH microbes and ileal BSH activity in mice with ASH; Figure S4: BHP treatment enhanced fecal BSH activity in mice with ASH; Figure S5: BHP significantly reduces serum LPS levels in alcohol-exposed mice; Figure S6: The inhibition of intestinal FXR activation abolished the protective effects of BHP on hepatic FXR signal.

## Abbreviations

ASH, alcoholic steatohepatitis; BHP, polysaccharides from *Balanophora harlandii* Hook.; BAs, bile acids; FXR, farnesoid X receptor; CYP7A1, cholesterol 7-alpha hydroxylase; NTCP, sodium taurocholate cotransporting polypeptide; FGF15, fibroblast growth factor 15; ASBT, apical sodium-dependent bile acid transporter; Fex, Fexaramine; Gly-MCA, Glycine-muricholic Acid; LC-MS, liquid chromatograph-mass spectrometer; SHP, small heterodimer partner; BSEP, bile salt export pump; BSH, bile salt hydrolase; T-α-MCA, tauro-α-muricholic acid; α-MCA, α-muricholic acid; LCA, lithocholic acid; HDCA, Hyodeoxycholic acid; CDCA, Chenodeoxycholic acid; TUDCA, tauroursodeoxycholic acid; DCA, deoxycholic acid; DHCA, Dehydrocholic acid; CA, cholic acid; MCA, muricholic acid; TCA, taurocholic acid; GDCA, Glycodeoxycholic acid; TLCA, Taurolithocholic acid.
