# Supplementary material for: The Polysaccharides from Balanophora harlandii Hook. Alleviated Alcoholic Steatohepatitis via Suppressing Bile Acid Synthesis Mediated by Intestinal FXR Activation
Source: Nutrients. 2026 Sep 3;18(17):2897. doi: 10.3390/nu18172897 (PMC13566957; doi:10.3390/nu18172897)
Supplement: Supplementary file 1 [file nutrients-18-02897-s001.zip › nutrients-4394793-supplementary.pdf]

Supplementary Table S1 Primers

Oligo Name	Sequence(5'to3')
GAPDH-F	AACTTTGGCATTGTGGAAGG
GAPDH-R	ACACATTGGGGGTAGGAACA
TNF- α -F	CCTGTAGCCCACGTCGTAG
TNF- α -R	GGGAGTAGACAAGGTACAACCC
IL-1 β -F	GAAATGCCACCTTTTGACAGTG
IL-1 β -R	TGGATGCTCTCATCAGGACAG
iNOS-F	GTTCTCAGCCCAACAATACAAGA
iNOS-R	GTGGACGGGTCGATGTCAC
MCP-1-F	TTAAAAACCTGGATCGGAACCAA
MCP-1-R	GCATTAGCTTCAGATTTACGGGT
SREBP1c-F	GCAGCCACCATCTAGCCTG
SREBP1c-R	CAGCAGTGAGTCTGCCTTGAT
F4/80-F	GCCTTCTGGATCCATTTGAA
F4/80-R	TGCATCTAGCAATGGACAGC
Fasn-F	TGGGTAATCCATAGAGCCCAG
Fasn-R	GGAGGTGGTGATAGCCGGTAT
Cyp7A1-F	GGTATGGAATCAACCCGTTGTC
Cyp7A1-R	GGGATTGCTGTGGTAGTGAGC
FXR1-F	AGGGGTTAGACAGCTCAGAATTT
FXR1-R	CCAGAGGGCCTGAAAGAGAGA
Occludin-F	CCGGATAAAAAGAGTACGCTGG
Occludin-R	TTGAAAGTCCACCTCCTTACAGA

Supplementary Figure:

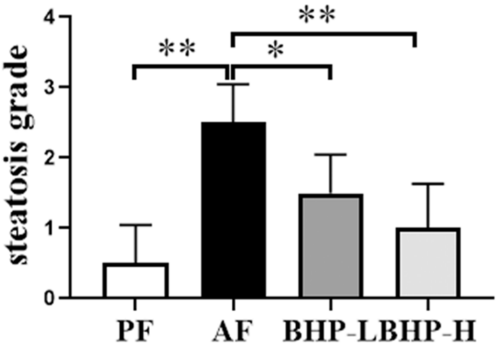

Figure S1: BHP treatment significantly reduces the degree of hepatic steatosis in mice exposed to alcohol. Data are presented as mean ± SEM (n = 5). PF, paired feed; AF, alcohol feed; BHP-L, AF + BHP-L; BHP-H, AF + BHP-H.* $p < 0.05$; ** $p < 0.01$.

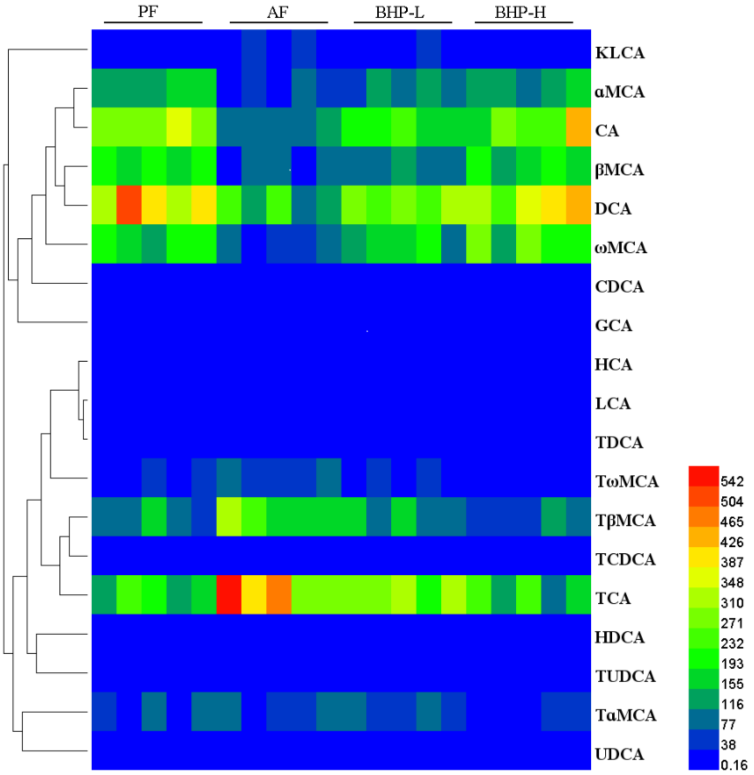

Figure S2: Heatmap of bile acid levels in mice ileal content. Data are presented as mean ± SEM (n = 5). PF, paired feed; AF, alcohol feed; BHP-L, AF+BHP-L; BHP-H, AF+BHP-H.

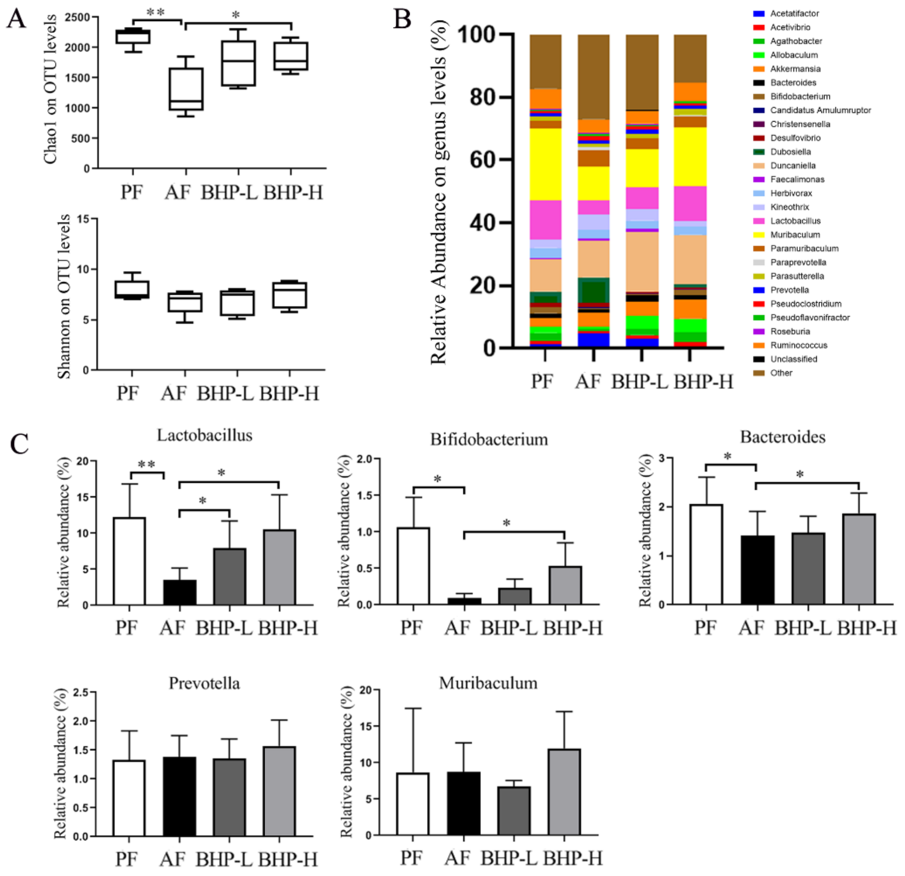

Figure S3: BHP treatment increased the abundance of BSH microbes and ileal BSH activity in mice with ASH. A) Chao1 and Shannon indices on OTU levels; B) relative abundance of gut bacteria at the genus level; C) relative abundance of high BSH-expressing microbes at the genus level. Data are presented as means ± SD ($n = 5$). PF, paired feed; AF, alcohol feed; BHP-L, AF + BHP-L; BHP-H, AF + BHP-H; * $p < 0.05$; ** $p < 0.01$.

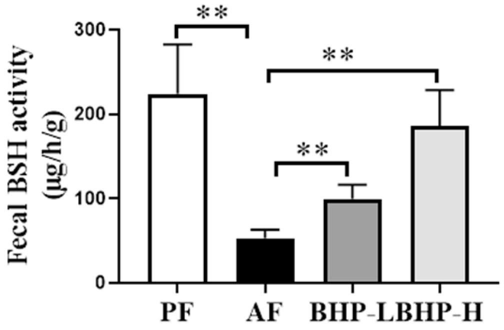

Figure S4: BHP treatment enhanced fecal BSH activity in mice with ASH. Data are presented as means ± SD ($n = 5$). PF, paired feed; AF, alcohol feed; BHP-L, AF + BHP-L; BHP-H, AF + BHP-H; ** $p < 0.01$.

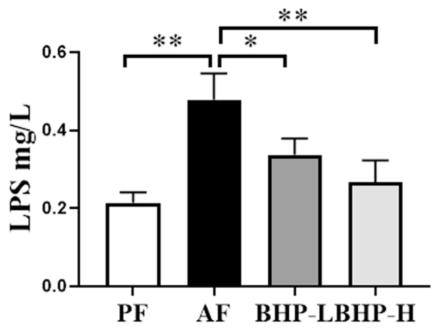

Figure S5: BHP significantly reduces serum LPS levels in alcohol-exposed mice. PF, paired feed; AF, alcohol feed; BHP-L, AF+BHP-L; BHP-H, AF+BHP-H; * $p < 0.05$; ** $p < 0.01$.

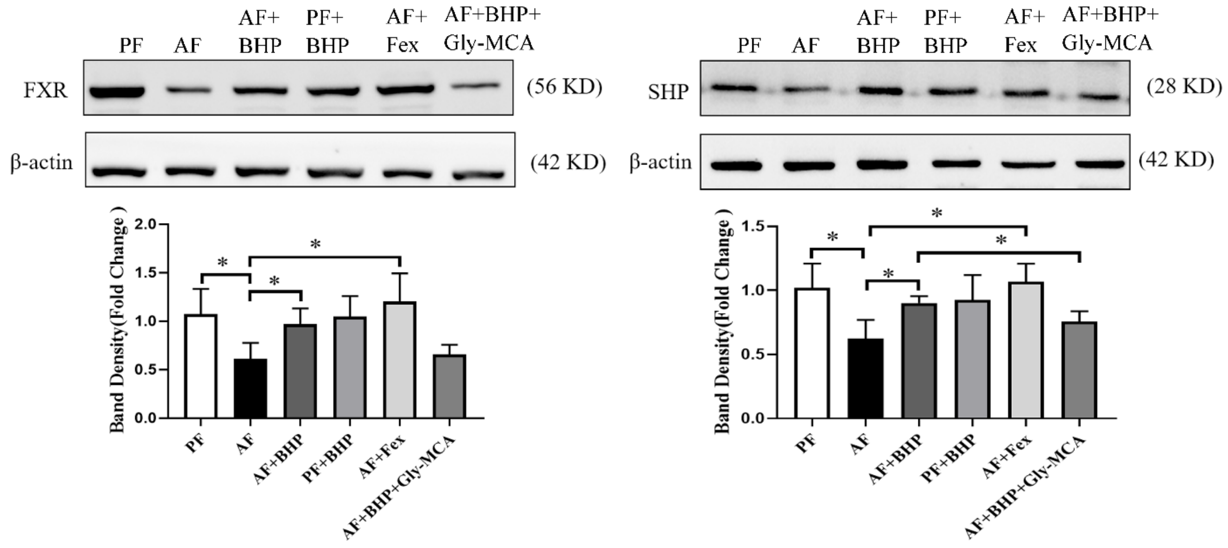

Figure S6: The inhibition of intestinal FXR activation abolished the protective effects of BHP on hepatic FXR signal. Data are expressed as mean ± SD ($n = 8$). PF, paired feed; AF, alcohol feed; Fex, Fexaramine; Gly-MCA, glycine-β-muricholic acid. * $p < 0.05$.
